# Ultrasound application for the decontamination of roselle (*Hibiscus sabdariffa* L.) seeds: Influence on fungal inhibition and seed quality

**DOI:** 10.1016/j.ultsonch.2023.106404

**Published:** 2023-04-10

**Authors:** Aminallah Tahmasebi, Ashkan Asgari, Somayeh Bakhshi, Amir Ghaffar Shahriari, Chul Won Lee

**Affiliations:** a Department of Agriculture, Minab Higher Education Center, University of Hormozgan, Bandar Abbas, Iran; b Plant Protection Research Group, University of Hormozgan, Bandar Abbas, Iran; c Research Group of Agroecology in Dryland Areas, University of Hormozgan, Bandar Abbas, Iran; d Department of Plant Protection, College of Agriculture, Shiraz University, Shiraz, Iran; e Department of Agriculture and Natural Resources, Higher Education Center of Eghlid, Eghlid, Iran; f Department of Chemistry, Chonnam National University, Gwangju, Republic of Korea

**Keywords:** Biochemical defense mechanism, *Fusarium solani*, Roselle, Seed decay, Ultrasound treatment

## Abstract

Seed decay is a major problem caused by pathogens that adversely affect seed yield and quality in agricultural production. Herein, the effect of 28 KHz ultrasound treatment for 20, 40 and 60 min and 1.5% sodium hypochlorite solution for 20 min was assessed for the decontamination of roselle (*Hibiscus sabdariffa* L.) seeds. In addition, seed germination indices, seedling growth traits, total phenolic content and the activity of defense-related enzymes, viz. peroxidase, superoxide dismutase, catalase and malondialdehyde were measured in the treated seeds. An isolate of *Fusarium solani* was obtained from roselle seeds and identified as the causal agent of roselle seed rot based on morphological and molecular characteristics. After six days of seed storage, the microbial infection caused the highest seed rot in the control seeds on the average of 56.67%, whereas ultrasound treatment for 60 min could remarkably reduce the seed decay by 3.33%. At the end of seed storage, the fungal load showed the highest (7.72 Log CFU ml^−1^) and lowest (6.99 Log CFU ml^−1^) rates in the control and ultrasound treatment for 60 min, respectively. Total phenolic content was significantly increased in ultrasound treatment for 60 min compared to control and sodium hypochlorite treatments. Moreover, the activity of peroxidase, superoxide dismutase and catalase was noticeably improved in ultrasound treatment for 60 min. Furthermore, ultrasound treatment did not show any adverse effects on seed germination indices and seedling growth traits of the roselle plants. Overall, ultrasound treatment for 60 min could effectively decrease roselle seed decay and the fungal load without changing seed and seedling quality.

## 1 Introduction

Roselle (*Hibiscus sabdariffa*; Malvaceae) is a tropical herb employed in traditional medicine with various therapeutic uses. Additionally, the roselle seed is an excellent source of dietary fiber [1]. Previous phytochemical and pharmacological studies have also confirmed *H. sabdariffa* properties with a wide range of nutritional and health benefits [2]. Different parts of roselle are also used for various food purposes around the globe [3]. *H. sabdariffa* is attacked by a variety of plant pathogens in the field and during storage conditions. Seed rots are one of the most important diseases that cause significant damage to the plant and limit roselle production by reducing seed viability and seedling vigor [4], [5]. Seed- borne diseases decrease seed quality and yield which may reduce consumers’ inclination toward utilizing this type of seeds [6]. It has also been shown that mold infection reduces the contents of seed storage reserves [7]. Seed diseases are responsible for losing more than 40% of the total yield of harvested seeds [8]. In addition, contaminated seeds might be regarded as sources of fungal inoculums to spread further infection. *Fusarium* is a fungal genus that could cause various plant diseases with severe economic damage [9]. A vast number of crops are infected and damaged as a result of *Fusarium* species infection [10]. It has been demonstrated that *Fusarium solani* infection results in roselle wilt and limits roselle production [11]. It has also been shown that *F. solani* is the dominant pathogen causing the roselle wilt disease in Taiwan [10]. *Fusarium* species persist in soil for several years by producing chlamydospores [9] and survive in harsh conditions [12]. As a result, there are limited effective ways to control *Fusarium* species infection [10]. Over the last decades, synthetic chemicals have been extensively used to manage seed-borne diseases [13], [14], [15], [16]. The application of systemic fungicides is considered a routine strategy to manage diseases and decontaminate seeds from fungal infections [13]. However, the off-target effects of fungicide treatment on seed microbiome and soil microbes and its adverse effects on soil and environmental problems have raised some concerns [17], [18], [19], [20]. Therefore, nowadays, much willingness has been paid to eco-friendly ways of controlling seed-borne diseases. There is also an increasing demand for new, safe and effective methods for seed decontamination. Ultrasound is a shape of vibrational energy produced by sound waves [21] and is vastly applied as a promising technology to control food pathogens and preserve fresh fruit and vegetable products [22], [23], [24], [25], [26]. This technology is considered an eco-friendly and safe technique and widely used for inactivating pathogens and decreasing the microbial content in food products [27]. Ultrasound has also extensively been applied as an effective priming method for breaking the dormancy of seeds and improving seed germination indices and seedling growth [28], [29], [30]. Considering the excellent nutritional and therapeutic sources of roselle seeds and the detrimental effects of fungal infection on seed productivity and quality, effective and safe techniques are required to decontaminate roselle seeds. The use of ultrasound to decontaminate seeds increases agricultural production both economically and environmentally because it eliminates the need for fungicides. Moreover, studies on food products have verified the ultrasound method's efficacy and safety. However, there is limited information regarding the effects of ultrasonic technology on microbial decontamination of seeds. Therefore, this study aimed to investigate the impact of ultrasound treatments duration of 20, 40 and 60 min on the decontamination of roselle seeds and its influence on seed and seedling quality.

## 2 Materials and methods

### 2.1 Plant materials and treatments

Roselle seeds were collected from a field in Minab, Iran in April 2021 and kept dry in the dark at 4 °C. Seeds were subjected to distilled water (control), sodium hypochlorite 1.5% for 20 min, ultrasound treatment for 20 min, 40 min and 60 min. Regarding ultrasound treatments, roselle seeds were immersed in a water bath ultrasonic cleaning chamber (Parsonic 15 s, Pars Nahand Engineering Company, Iran) containing sterile water at the frequency of 28 KHz. Then, the treated seeds were placed on sterile filter papers to be dried.

### 2.2 Rot rate, seed germination indices and seedling growth traits

Roselle seeds were placed on filter paper in Petri dishes, sealed with parafilm and stored at room temperature. Rot rate and germination indices of roselle seeds were recorded every day until 6 days after placing the seeds in Petri dishes. The seeds with about 2 mm radicle length were considered germinated ones. Seedling growth traits (plant shoot and root length, shoot and root fresh weight, shoot and root dry weight) were assessed six days after placing the seeds in Petri dishes. A number of 10 seeds were selected for each replicate. In the second experiment, seeds were cultured in trays and kept in a growth chamber at 24 ± 2 °C, relative humidity of 65% with an 18-h photoperiod. Seed germination indices and seedling growth traits (plant shoot and root length, shoot and root fresh weight, shoot and root dry weight) were checked on the tenth day after culturing the seeds in trays. A number of six seeds were selected for each replicate. Shoot and root dry weight was measured after drying at 65 °C for 12 h. Germination percentage (GP), germination rate index (GRI), mean germination time (MGT), emergence percentage (EP), mean emergence time (MET), emergence rate index (ERI) and vigor index (VI) were calculated based on the following formula [31], [32], [33]:GP = (N/M) * 100

N and M represent the total number of germinated and cultivated seeds after seven days.GRI or ERI = (G1/1) + (G2/2) + …+ (Gx/x)

G1 and G2 show the germination percent in the first and second days to the final day,MGT or MET = ∑Dn / ∑n

where n represents the number of germinated seeds or emerged seedlings on day D and Dn shows the number of days counted since the germination experiment began.GI = (7 * N1) + (6 * N2) + (5 * N3) + …

N1, N2, … = the number of germinated seeds in the first day, second day and …,VI = GP × seedling length (SL)EP = (E/N) * 100

E and N are the total numbers of emerged seedlings and seeds.ERI = (E1/1) + (E2/2) + …+ (Ex/x)

E1 and E2 represent the number of emerged seedlings in the first and second count days to the final count day.

### 2.3 Identification of causal agent of seed rot

In order to lessen contaminants, sodium hypochlorite (1%) was used to sterilize roselle seeds for 10 min. After washing three times with sterile water, the roselle seeds were plated on potato dextrose agar (PDA), incubated at 25 °C for six days in a 12-h photoperiod and subcultured on PDA. A portion of the fungal mycelium was placed on the tube bottom and the seeds were put above the fungus inoculum. Fungal mycelium covered the roselle seeds after eight days and caused seed rot. Morphological characterization of the fungal isolate was performed [34], [35]. After seven days of incubation, colony features on PDA were recorded and colony pigments were determined according to color chart [36]. Microscopic observations revealed the presence of macroconidia, microconidia, sporodochia, phialide, false heads and chlamydospores from colonies cultivated on CLA (Carnation Leaf-piece Agar), SNA (Spezieller Nährstoffarmer Agar) and KCl agar. Morphological identification of the fungus isolate was confirmed by amplification and sequencing of the ITS-rDNA genomic region. Total DNA was extracted from fresh mycelium grown on PDA (25 °C for five days), using the protocol previously described [37]. Polymerase chain reaction (PCR) amplification of ribosomal DNA-based ITS1-5.8S-ITS2 region was conducted using the universal ITS1 and ITS4 primers [38] in a Thermocycler device (BIORAD, T100-Thermal cycler). PCR was performed in 25-μL reactions and each reaction consisted of template DNA (50 ng/μL), each primer (0.4 μM), 10 μl of 2 × Master Mix (Ampliqon, Denmark) and 11 µl deionized water. The PCR program conditions were initial denaturation at 95 °C for 5 min, 95 °C for 40 s (30 cycles), 58° C for 40 s, 72 °C for 40 s, and a final extension at 72 °C for 10 min. PCR products were visualized on agarose gel (1% (w/v)) and the amplicon presence and size were confirmed. Amplification products were purified and sequenced by Cardiogenetic Research Center (Tehran, Iran). The obtained sequence was deposited in the GenBank database.

### 2.4 Total phenolic content

To determine the total phenol content, 100 μl of seed extract (1 mg/ml, 80% methanol), 100 μl of sodium carbonate solution (2.5%) and 20 μl of Folin Ciocalteus (50%, water) were completely mixed, vortexed and incubated for 30 min. The absorbance values of seed treatments were determined with the spectrophotometer (DR3900, Hach, Germany) at 750 nm and total phenol concentrations were determined based on the gallic acid standard curve.

### 2.5 Enzyme assays

Seed samples (1 g) were frozen in liquid nitrogen and ground in 10 ml extraction buffer (0.1 M phosphate buffer, pH 7.5 with 0.5 mM EDTA). Then, the samples were filtered and centrifuged for 20 min at 15,000 g at 4 °C. The supernatant was used to measure the enzymatic activities of superoxide dismutase (SOD), peroxidase (POD), catalase (CAT) and malondialdehyde (MDA) [39]. CAT activity was spectrophotometrically evaluated at 240 nm by measuring a decrease in H_2_O_2_ absorbance [40]. The amount of 0.5 ml H_2_O_2_ (75 mM) was added in 50 µl enzyme extract and 1.5 ml phosphate buffer (0.1 M; pH 7) and 0.95 ml distilled water. The decrease in H_2_O_2_ absorbance was measured at 240 nm for 2 min. POD activity was determined spectrophotometrically with guaiacol as the substrate at 470 nm [40]. The amounts of 1 ml phosphate buffer (0.05 M, pH 6.1), guaiacol 0.5 ml (92 mM), 0.5 ml H_2_O_2_ (18 mM), 100 µl enzyme extract and 900 µl distilled water consisted the reaction mixture. SOD activity was measured as its ability of 50% inhibition of nitro blue tetrazolium chloride reduction at 560 nm [41]. The reaction mixture included methionine (200 mM), sodium carbonate (1.5 M), EDTA (3.0 mM), nitro blue tetrazolium chloride (2.25 mM), riboflavin (60 mM), and phosphate buffer (100 mM, pH 7.8). Malondialdehyde concentration was measured using extraction methods [42]. Seed samples were homogenized in trichloroacetic acid solution, centrifuged at 12,000 g for 5 min and the supernatants were collected. The reaction mixture (0.5 ml seed extract, 1 ml trichloroacetic acid (20%) containing 0.5% thiobarbituric acid) was kept in boiling water for 30 min, cooled on ice and centrifuged at 10,000 g for 10 min. The absorbance values of seed treatments were measured at 532 and 600 nm by spectrophotometer (DR3900, Hach, Germany).

### 2.6 Fungal quantification

A solution was prepared from treated and control seeds with three replications. One seed from each treatment was placed in 20 ml Potato dextrose broth culture and shaken for 24 h. Then, the number of *F. solani* with colony forming units (CFU) colonizing the roselle seed was quantified by plating 20 µl from a serial dilution of treated and control seed suspensions (1/10, 1/100, 1/1000 and 1/10000) onto Petri plates containing the PDA medium. The plates were kept at 25 °C for 7 d and colonies were counted to determine the number of *F. solani* in triplicate for the seed samples. The colonies were transferred to PDA plates and the presence of *F. solani* was checked based on the morphological characteristics to confirm *F. solani* infection. After counting the colonies, the number of *F. solani* was expressed as Log CFU ml^−1^ based on the following equation:CFU/ml = number of colonies / dilution * ml plated suspension

### 2.7 Statistical analysis

One-way analysis of variance (ANOVA) of data was conducted and then the significant differences among treatments were performed by Duncan’s multiple range test using SPSS (version 21) at *p* < 0.05 level of confidence. The experiment was based on a completely randomized design with three replications. Pearson correlation, principal component and cluster analysis were done using SPSS and Minitab software.

## 3 Results and discussion

### 3.1 Identification of causal agent of seed rot and its infection rate in roselle seeds

**Fig. 1:**
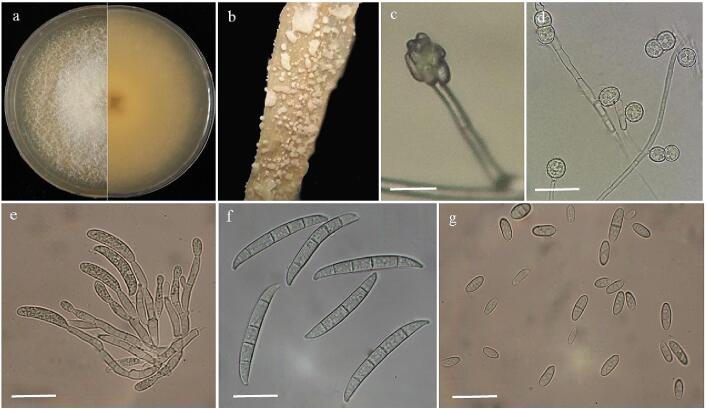
Morphology of *Fusarium solani* isolated from roselle seeds. a, Colony on PDA after five days at 25 °C in the dark, top and reverse side view. b, Sporodochia on carnation leaf in CLA. c, Phialide. d, Chlamydospores. e, Sporodochial conidiophores. f, Macroconidia. g, Microconidia. Scale bars c, e = 50 μm; all others = 20 μm.

**Fig. 2:**
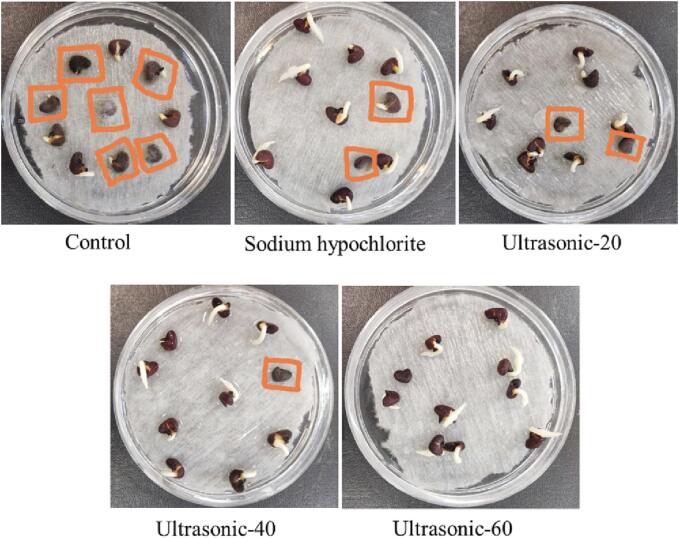
Photographs of control seeds and seeds subjected to sodium hypochlorite 1.5% for 20 min, ultrasound treatment for 20 min, 40 min and 60 min and their effects on seed rot after three days of storage at room temperature. The rotten seeds in each treatment were shown by pink color.

**Fig. 3:**
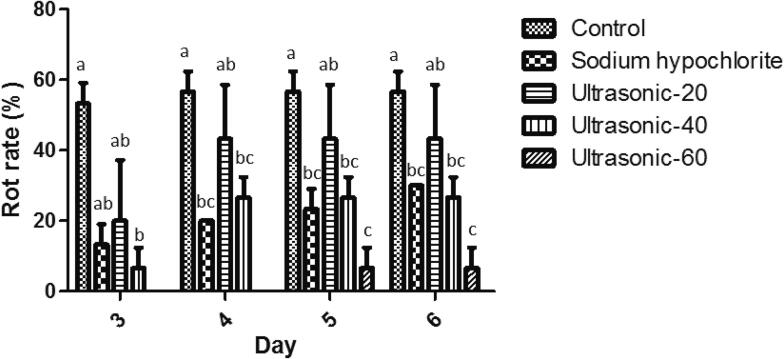
Effect of seed treatments on the *F. solani* rot rate of roselle seeds.

Based on the morphological (Fig. 1) and molecular characteristics, *F. solani* isolate HSS was identified*.* The top and reverse sides of mycelium showed creamy or white and brownish cream to purple colonies on PDA after one week. Aerial mycelium showed white and cottony mycelium on PDA. Conidiophores formed on mycelium were long, straight, simple or branched. Cream color sporodochium was abundantly formed on carnation leaf agar medium for 10 days under 12/12 h light /dark. Macroconidial septation was from 3 to 6 (mostly 4-septate). The dorsal side of the macroconidia arising from sporodochia was more curved than the ventral side. Their first and final cells showed papillate and blunt apical cells and foot-shaped basal cells. The microconidia were oval, reniform and obovoid with mostly one and sometimes two-septate conidia. Chlamydospores were abundantly formed in mycelium, globose, subglobose, intercalary or terminal and rough walled, singly or in chains with 5–15 μm in diameter. Sequence analysis of the ITS-1 region revealed 99.61% nucleotide identity of the isolate HSS (GenBank accession no. OP268363.1) with the *F. solani* isolate KHK2 (MH559638.1) from India. Herein, we report *F. solani* as the causal agent of seed rot on roselle seeds in Iran (Fig. 2). In our study, ultrasound treatment for 60 min could significantly decrease roselle seed decay (Fig. 3). Seed rot rate was decreased until 3.3% in the treated seeds using ultrasound treatment for 60 min at six days after storing seeds, while it was 56.67%, 43.33%, 26.67% and 23.33% for the control treatment and treated seeds by ultrasound treatment for 20 min, ultrasound treatment for 40 min and sodium hypochlorite, respectively (Fig. 3). It has also been shown that *F. solani* could infect other parts of roselle and cause root rot, wilting and rotted pith in roselle [10], [43] which may negatively affect plantings, production, yield and quality of roselle.

### 3.2 Seed germination indices and seedling growth traits

Seed germination indices (GP, GRI, MGT, EP, MET, ERI and VI) and growth traits (plant shoot and root length, shoot and root fresh weight, shoot and root dry weight) showed no significant difference between control and other treatments (Table 1, Table 2). Therefore, ultrasound treatment for 60 min did not show any adverse effects on seed germination and seedling growth traits of the roselle plants. In another study, ultrasonic application increased the germination of aged seeds and seedling growth [30], [44]. However, no significant positive or negative effect of ultrasound irradiation was found on the growth of tomato and rice plants [45]. It has also been demonstrated that ultrasound application could preserve food's nutritional and sensory quality and reduce microbial contamination [21]. The previous study showed that ultrasound temperature was the main factor for aged seeds germination [44]. Regarding the extraction and yield of bioactive compounds through the ultrasound approach, ultrasound effects depend on the complexity and diversity of plant structures and the plant/material used [46], [47]. Other factors including ultrasound intensity and frequency, the processing time, the type, concentration, and pH of the solvent could influence the efficiency of ultrasound-assisted extraction [48], [49]. These parameters may explain the different ultrasound effects on seed germination and seedling growth in the various studies. Therefore, we could suggest ultrasound treatment for 60 min a safe and effective method for the control of roselle seed decontamination. However, further studies are required to evaluate the effects of other parameters on the efficacy of ultrasound approach in the decontamination of roselle seed.

**Table 1:** Effect of seed treatments on the germination parameters and seedling growth traits of roselle (*Hibiscus sabdariffa* L.) seeds.

Sources of variations (S.O.V)	Germination Percentage	Germination Rate Index	Mean Germination Time	Root Length (mm)	Shoot length (mm)	Root Fresh weight (g)	Shoot Fresh Weight (g)	Root Dry Weight (g)	Shoot Dry Weight (g)	Vigor Index
Treatment	140 ^ns^	1.125 ^ns^	0.00081 ^ns^	17.19 ^ns^	29.02 ^ns^	0.00002 ^ns^	0.00021 ^ns^	3.34 E-7 ^ns^	0.000002 ^ns^	1669535 ^ns^
Error	120	1.083	0.00440	11.80	22.51	0.00006	0.00044	1.10 E-7	0.000003	1,203,280
CV	12.73	12.49	6.26	15.99	13.19	20.64	11.85	17.57	7.55	22.00

Data were subjected to analysis of variance and ns shows the non-significant differences among the treatments at *p* < 0.05 level of confidence.

**Table 2:** Effect of seed treatments on the emergence parameters and seedling growth traits of roselle (*Hibiscus sabdariffa* L.) seedlings.

Sources of variations (S.O.V)	Emergence Percentage	Emergence Rate	Mean Emergence Time	Root Length (mm)	Shoot length (mm)	Root Fresh weight (g)	Shoot Fresh Weight (g)	Root Dry Weight (g)	Shoot Dry Weight (g)	Vigor Index
Treatment	259.25 ^ns^	0.30 ^ns^	0.23 ^ns^	50.55 ^ns^	25.80 ^ns^	0.00025 ^ns^	0.0035 ^ns^	0.000012 ^ns^	0.00013 ^ns^	4384482 ^ns^
Error	444.44	0.98	0.17	10.52	11.05	0.00015	0.0033	0.000005	0.00007	6,836,107
CV	27.90	32.82	21.69	5.61	5.10	9.47	14.30	19.73	18.33	28.13

Data were subjected to analysis of variance and ns shows the non-significant differences among the treatments at *p* < 0.05 level of confidence.

### 3.3 Total phenolic content

**Fig. 4:**
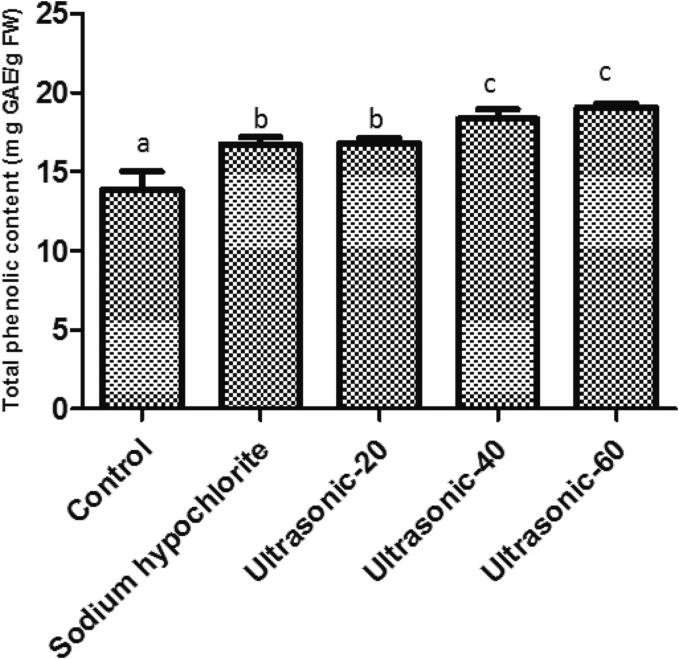
Effect of seed treatments on the total phenolic content of roselle (*Hibiscus sabdariffa* L.) seeds.

Ultrasound treatment for 40 and 60 min significantly improved the total phenolic content of roselle seeds (Fig. 4). It has been shown that phenolic acids are involved in seed resistance to microbial decay [50]. The defensive role of phenolic compounds in plants is mainly related to their antioxidant activities and scavenging free radicals from lipid peroxidation which causes reactive oxygen species inhibition [51], [52]. Moreover, phenolic chemicals are crucial for the induction of plant resistance [53]. In another study, aerial ultrasound application induced disease resistance in tomato and rice against *Fusarium* wilt and blast diseases [45]. Therefore, we can conclude that ultrasound application for 40 and 60 min could induce roselle seed resistance against *F. solani*, possibly through induction of seed defense mechanisms like increased total phenolic content and enzyme activity. In another study, it has been suggested that salicylic acid (SA) signaling pathway may be an ultrasound mechanism in plant resistance against diseases [45]. Sound irradiation could prime systemic acquired resistance and upregulate expression of SA signaling pathway-related genes in *Arabidopsis thaliana* against *Botrytis cinerea* infection [54]. However, we cannot rule out the possibility that physical and chemical effects caused by ultrasound waves may play a role in the elimination of fungal contamination of roselle seeds.

### 3.4 Enzyme activity

**Fig. 5:**
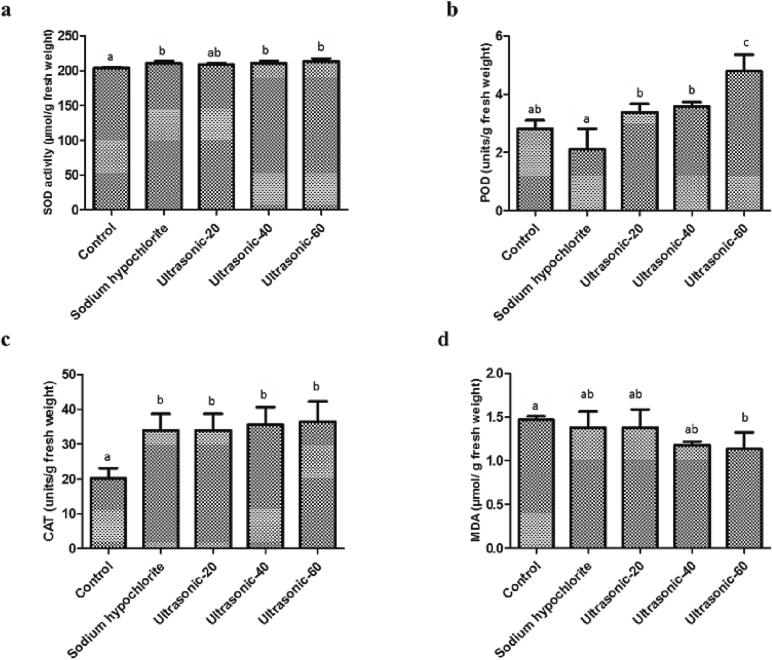
Effect of seed treatments on the enzyme activities (superoxide dismutase (a), peroxidase (b), catalase (c) and malondialdehyde (d)) of roselle (*Hibiscus sabdariffa* L.) seeds.

The activity of peroxidase, superoxide dismutase and catalase was noticeably improved in ultrasound treatment for 60 min (Fig. 5). However, MDA activity was significantly decreased in the treated seeds with ultrasound treatment for 60 min. Our results were consistent with another study which showed that ultrasound treatment refreshes aged grass seeds by increasing SOD and POD activities and reducing MDA content [44]. It has also been shown that ultrasonic treatments reduced MDA activity in aged tall fescue (*Festuca arundinacea*) and Russian wild rye (*Psathyrostaehys juncea*) seeds [30]. The reduction in MDA content might be attributed to the ultrasound effect on inactivating the enzymes like lipooxygenase in seeds [30]. SOD and POD activities were increased in the treated seeds with ultrasound [30], which may prevent the accumulation of reactive oxygen species [55]. The MDA is generated at the final stage of lipid peroxidation and excessive reactive oxygen species may accelerate the peroxidation of lipids and deteriorate seeds during storage [56]. It has also been shown that polyphenol oxidase (PPO) as enzyme-based biochemical defense is involved in seed resistance of wild oat (*Avena fatua* L.) to seed-decaying *Fusarium* fungi [57]. Induction of defense enzymes, including PPO and POD have been reported in wheat against *Fusarium graminearum* [58], [59]. Our results are in accordance with the hypothesis that seeds contain fundamental inducible enzyme-based biochemical defense mechanisms to protect seed survival and longevity [57]. Seeds have both physical and chemical defenses to protect food reserves against pathogens and herbivores [60]. There have been previous reports of the functions of enzymes in seed defense [57], [61]. Enzymatic antioxidants like CAT, SOD and POD break down and remove free radicals and prevent seed from deterioration [62].

### 3.5 Fungal analysis

**Fig. 6:**
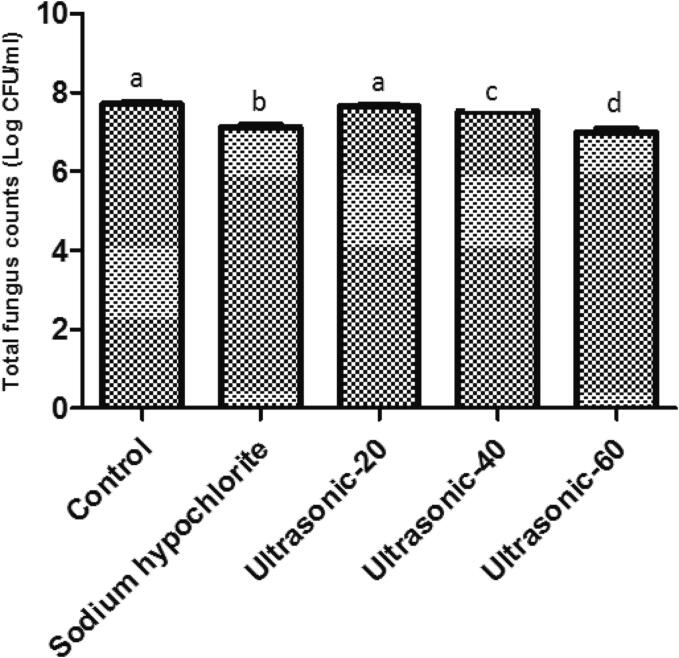
Effect of seed treatments on the fungal load (Log CFU ml^−1^) of roselle (*Hibiscus sabdariffa* L.) seeds.

At the end of seed storage, the fungal load showed the highest (7.72 Log CFU ml^−1^) and lowest (6.99 Log CFU ml^−1^) rates in the control and ultrasound treatment for 60 min, respectively. Compared with the control group and other treatments, ultrasound treatment for 60 min significantly decreased total fungal counts (*p* < 0.05). There was no significant difference in the total fungus counts between the control group and ultrasound treatment for 20 min (Fig. 6). Compared to ultrasound treatment for 20 and 40 min, the treatment duration of 60 min could strongly inhibit *F. solani* fungus as the causal agent of roselle seed decay. It has been demonstrated that several factors including ultrasound exposure time could increase the fungal inactivation level [63]. Antimicrobial activity of the ultrasonic approach might be attributed to intracellular acoustic cavitation which could increase the membranes permeability, thinning of cell membranes, free radicals generation and localized heating [64], [65], [66]. Shock waves caused by ultrasound technology could also break the cell wall and membrane structures of microbes and inactivate microbial key enzymes resulting in a disruption in microbial cells and cell death [66], [67]. Chemical effect is caused by reactive compounds and free radicals [68]. Therefore, the physical and chemical effects of ultrasound application are involved in microbial inactivation [69]. The application of ultrasonic waves could be a promising way to reduce the counts of total fungus on roselle seeds. Previously, it has also been shown that ultrasound technology reduced the content of mold fungi in the grain [70]. Furthermore, high-power ultrasound could completely inhibit yeasts and molds growth in fruit juices and nectars [71]. Additionally, aerial ultrasound decreased the rate of *Fusarium* wilt and blast diseases in tomato and rice [45]. Also, ultrasound application suppressed powdery mildew in strawberry [45]. Ultrasound treatment reduced the microbial load during green asparagus storage [72] and caused fungal inactivation and mycotoxin decontamination in food products [27]. Moreover, ultrasound application showed a significant effect on the pathogenic bacteria count in lettuce [73]. It has also been shown that ultrasound treatment could effectively inactivate *Escherichia coli*, *Salmonella Enteritidis*, *Listeria innocua* and *Staphylococcus aureus* infections on strawberries and lettuce [74]. Furthermore, high-intensity ultrasound effectively eliminated food-borne viruses [75].

### 3.6 Principal component analysis, cluster analysis and Pearson correlation

**Fig. 7:**
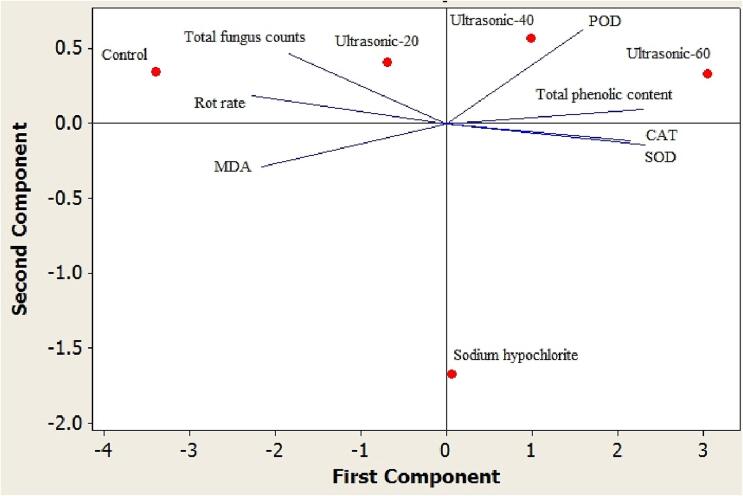
Biplot of the first two principal components for the rot rate, total fungus counts, total phenolic content and the activities of the enzymes, obtained by the five roselle seed treatments (control, sodium hypochlorite and ultrasound treatment for 20, 40 and 60 min).

**Fig. 8:**
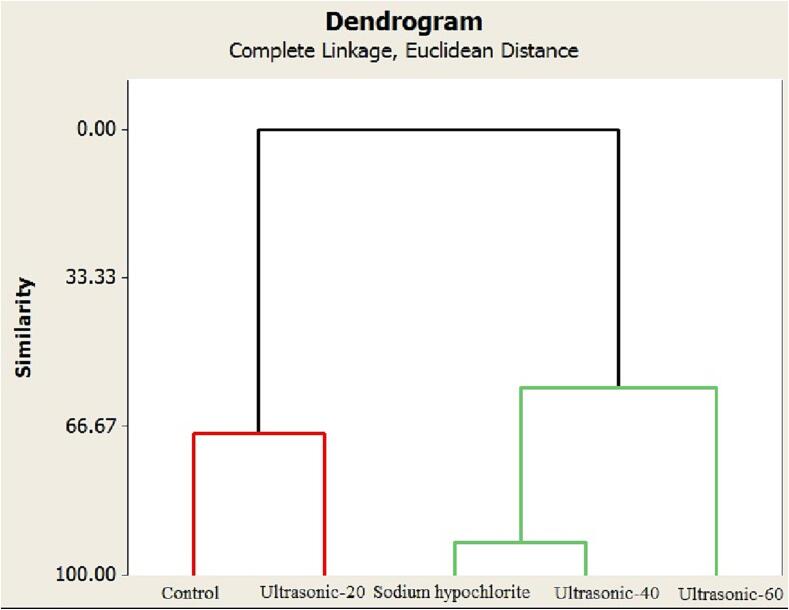
Hierarchical cluster analysis of the rot rate, total fungus counts, total phenolic content and the activities of the enzymes, obtained by the five roselle seed treatments (control, sodium hypochlorite and ultrasound treatment for 20, 40 and 60 min).

**Fig. 9:**
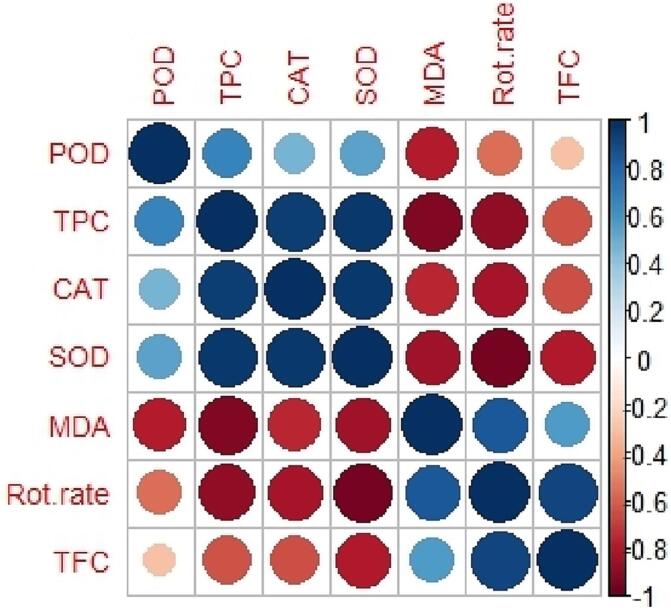
Corrplot between various measured characteristics of the five roselle seed treatments. POD (Peroxidase), TPC (Total phenolic content), CAT (Catalase), SOD (Superoxide dismutase), MDA (Malondialdehyde), TFC (Total fungus counts). Positive correlations are displayed in blue and negative correlations in red color. Color intensity and the size of the circle are related to the correlation coefficients.

Principal component analysis was conducted based on total fungus counts, rot rate, total phenolic content and the activity of the enzymes. The findings showed that according to the latter factors, the roselle seed treatments were placed into three different groups. Ultrasound treatment for 40 and 60 min was placed in a distinct group. Another group included the control group and ultrasound treatment for 20 min. The third group contained sodium hypochlorite treatment. Total phenolic content and the activities of the enzymes (CAT, POD and SOD) were placed in close proximity to the ultrasound treatment for 40 and 60 min (Fig. 7). It shows that these factors positively affect the potential of seed decontamination of ultrasound treatment for 40 and 60 min. Moreover, the hierarchical cluster analysis of the latter characteristics showed the similarity level among the roselle seed treatments (Fig. 8). The cluster showed the similarity range among the roselle seed treatments from 56.41% to 92.32%. Control group and ultrasound treatment for 20 min demonstrated 67.95% in total fungus counts, rot rate, total phenolic content and enzyme activity factors. Furthermore, sodium hypochlorite and ultrasound treatment for 40 min showed the highest similarity (92.32%), while these groups indicated 56.41% similarity with ultrasound treatment for 60 min. The findings of Pearson correlation analysis revealed a significant positive correlation between total fungus counts with rot rate in roselle seeds (r = 0 0.92). In addition, total phenolic content revealed a significant positive correlation with POD, CAT and SOD activities (r = 0.61, 0.78 and 0.81, respectively). Also, a significant positive correlation was found between CAT and SOD activity (r = 0.53). While, there was a significant negative correlation between total phenolic content and SOD activity with rot rate in roselle seeds (r =  − 0 0.88 and − 0.95, respectively). Also, a significant negative correlation was found between MDA activity with total phenolic content, POD and CAT activities (r =  − 0.66, −0.70 and − 0.58, respectively) (Table 3 and Fig. 9).

**Table 3:** Correlation coefficients between measured characteristics of the five roselle seed treatments.

	Rot rate	TFC	TPC	POD	CAT	SOD	MDA
Rot rate	1						
TFC	0.92*	1					
TPC	−0.88*	−0.63	1				
POD	−0.55	−0.30	0.61*	1			
CAT	-0.829	−0.64	0.78^**^	0.35	1		
SOD	−0.95*	−0.79	0.81^**^	0.36	0.53*	1	
MDA	0.84	0.56	−0.66^**^	−0.70^**^	−0.58*	−0.37	1

* Correlation is significant at the 0.05 level (2-tailed) and ** Correlation is significant at the 0.01 level (2-tailed). POD (Peroxidase), TPC (Total phenolic content), CAT (Catalase), SOD (Superoxide dismutase), MDA (Malondialdehyde), TFC (Total fungus counts).

## 4 Conclusion

Today's synthetic chemicals utilized in seed decontamination are hazardous to the environment and human health. Here, the effectiveness of applying ultrasound to reduce the fungal infection on roselle seed was investigated. A 60-minute ultrasound treatment could effectively lower the fungal infection in roselle seeds, leading to a significant decrease in seed deterioration. Additionally, when roselle seeds were subjected to ultrasonic for 60 min, their total phenolic content and defense enzyme activity (peroxidase, superoxide dismutase, and catalase) were increased. We could conclude that induction of seed defense mechanisms might be another ultrasound-based way to reduce total fungus counts. Moreover, seed germination indices and seedling growth traits did not exhibit any negative impacts after a 60-minute ultrasonic treatment. The results of this study reveal that ultrasound treatment for 60 min is a simple, efficient, time-saving, and environmentally friendly method that has the potential to be used to prevent fungal infection at a lower cost and without affecting seed quality.

## CRediT authorship contribution statement

**Aminallah Tahmasebi:** Conceptualization, Methodology, Formal analysis, Writing – original draft. **Ashkan Asgari:** Conceptualization, Data curation, Formal analysis. **Somayeh Bakhshi:** Methodology, Formal analysis. **Amir Ghaffar Shahriari:** Methodology, Resources. **Chul Won Lee:** Writing – review & editing.

## Declaration of Competing Interest

The authors declare that they have no known competing financial interests or personal relationships that could have appeared to influence the work reported in this paper.
